# Clinical efficacy study of a spherical nasal vestibular stent

**DOI:** 10.3389/fmed.2025.1748584

**Published:** 2026-01-13

**Authors:** Junxiao Jia, Jikuan Qiu, Rui Li, Baoshi Fan, Yi Lu, Xiangzhi Bai, Yu Song, Junxiu Liu

**Affiliations:** 1 Department of Otolaryngology-Head and Neck Surgery, Peking University First Hospital, Beijing, China; 2 Image Processing Center, Beihang University, Beijing, China; 3 Laboratory of Virtual Reality Technology and Systems, Beihang University, Beijing, China

**Keywords:** 3D reconstruction, CFD, clinical efficacy, single-arm trial, spherical nasal vestibular stent

## Abstract

**Objective:**

To design and validate a spherical nasal vestibular stent based on vestibular structural changes for treating nasal obstruction.

**Methods:**

This study enrolled 99 patients with nasal obstruction and confirmed positive findings on anterior rhinoscopy. Pre- and post-dilation sinonasal computed tomography (CT) scans were obtained until symptoms nearly resolved. Three-dimensional (3D) reconstruction was utilized to evaluate anatomical changes, and Spearman correlation analysis was performed to assess the relationship between these changes and visual analog scale (VAS) scores for nasal obstruction. Based on 3D reconstructed models and computational fluid dynamics (CFD) parameter evaluations, a nitinol mesh stent customized to the anatomical characteristics of the nasal vestibule was designed. A single-arm clinical trial in 31 patients was subsequently evaluated the stent using NOSE scores, acoustic rhinometry, and rhinomanometry before and after placement. Adverse events were systematically recorded.

**Results:**

3D reconstruction showed that changes in nasal vestibule volume before and after dilation correlated with patients’ VAS scores for nasal obstruction. In the clinical trial, the spherical nasal vestibular stent—designed using nasal vestibule volume data—significantly reduced nasal resistance (*p* < 0.05), increased nasal volume and valve area (*p* < 0.001), and lowered NOSE scores (p < 0.001). Most patients tolerated the stent well; side effects like dryness and pain were mild.

**Conclusion:**

Based on 3D models from dilated nasal vestibules, this study designed a spherical stent that effectively relieves nasal obstruction with minimal risk, positioning it as a promising non-surgical intervention for clinical application.

## Introduction

Nasal obstruction is a common manifestation of nasal pathology, significantly impairing quality of life and psychological well-being. The nasal vestibule accounts for 30–50% of total nasal resistance (1), indicating that structural alterations in this region have a critical impact on nasal ventilation. Anatomical abnormalities involving the nasal vestibule commonly affect both the internal and external nasal valves. The internal nasal valve is anatomically defined by the caudal edge of the upper lateral cartilage, the nasal septum, the lateral nasal wall, and the inferior turbinate (2). Management of internal nasal valve compromise is broadly categorized into surgical and non-surgical interventions. Surgical techniques—including alar batten grafts, lateral crural strut grafts, spreader grafts, butterfly grafts, and suspension sutures (3–7)—enhance airflow by reinforcing or reconstructing the structural integrity of the internal nasal valve. Non-surgical approaches include external and internal nasal dilators, such as Breathe Right strips (8, 9) and Nozovent silicone stents (10), which function through mechanical expansion of the internal nasal valve and stabilization of the nasal alae (11, 12). However, most current devices primarily target lateral dilation of the internal nasal valve, neglecting the potential advantages of simultaneously enlarging both the external and internal nasal valves—that is, increasing the overall volume of the nasal vestibule to optimize airflow dynamics. Moreover, clinical evidence supporting the efficacy and safety of these over-the-counter devices remains limited.

Based on 3D reconstruction models of the dilated nasal vestibule and CFD parameter analysis, this study proposes a novel spherical stent design for overall volume expansion. A nitinol spherical nasal vestibular stent was developed, and its efficacy and safety in alleviating nasal obstruction were systematically evaluated in clinical trials, offering new insights into non-surgical management.

## Method

### Study subjects

This study enrolled adult patients from the otolaryngology clinics of Peking University First Hospital and Peking University Third Hospital between July 2021 and October 2024. The protocol was approved by the ethics committees of both institutions.

Patients were included if they presented with persistent unilateral or bilateral nasal obstruction confirmed by a positive anterior rhinoscopy examination, had no history of rhinitis, no upper respiratory tract infection within two weeks prior to enrollment, no long-term use of nasal decongestants, and were not undergoing concurrent treatments for nasal obstruction. Exclusion criteria included pregnancy or lactation, nasal tumors, rhinosinusitis, hematological disorders, nitinol allergy, recent participation in other clinical trials, or any condition deemed unsuitable for the study by the investigators, including cognitive dysfunction that precluded completion of required assessment scales.

**Figure 1:**
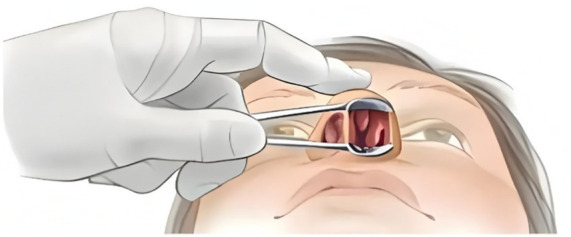
Anterior rhinoscopy test.

A positive anterior rhinoscopy test, similar to the modified Cottle maneuver (13), was defined as the patient reporting significantly improved nasal airflow after manually dilating the nasal valve and anterior naris with an anterior rhinoscope (Figure 1).

### Study protocol

#### Part 1: 3D reconstruction study before and after nasal vestibular dilation

After informed consent, eligible patients completed a VAS assessment. A sinonasal CT scan was performed under natural conditions (pre-dilation CT). The nasal vestibule was then dilated using an anterior rhinoscope until symptom relief, followed by a second CT scan (post-dilation CT), which was performed while patients self-maintained anterior nasal speculum insertion at a depth and angle that provided subjective relief of nasal obstruction. Prior to CT scan, participants were instructed on the proper technique for inserting and adjusting the speculum to achieve optimal dilation, followed by repeated practice sessions to ensure stability during scanning. DICOM data were imported into Mimics 21.0 for 3D reconstruction. The nasal airway was segmented by thresholding to generate a 3D model, which was exported in STL format for anatomical analysis and CFD validation. CFD were assessed using three key parameters: mean velocity, mean pressure, and mean wall shear stress.

#### Part 2: Clinical efficacy of the spherical nasal vestibular stent

**Figure 2:**
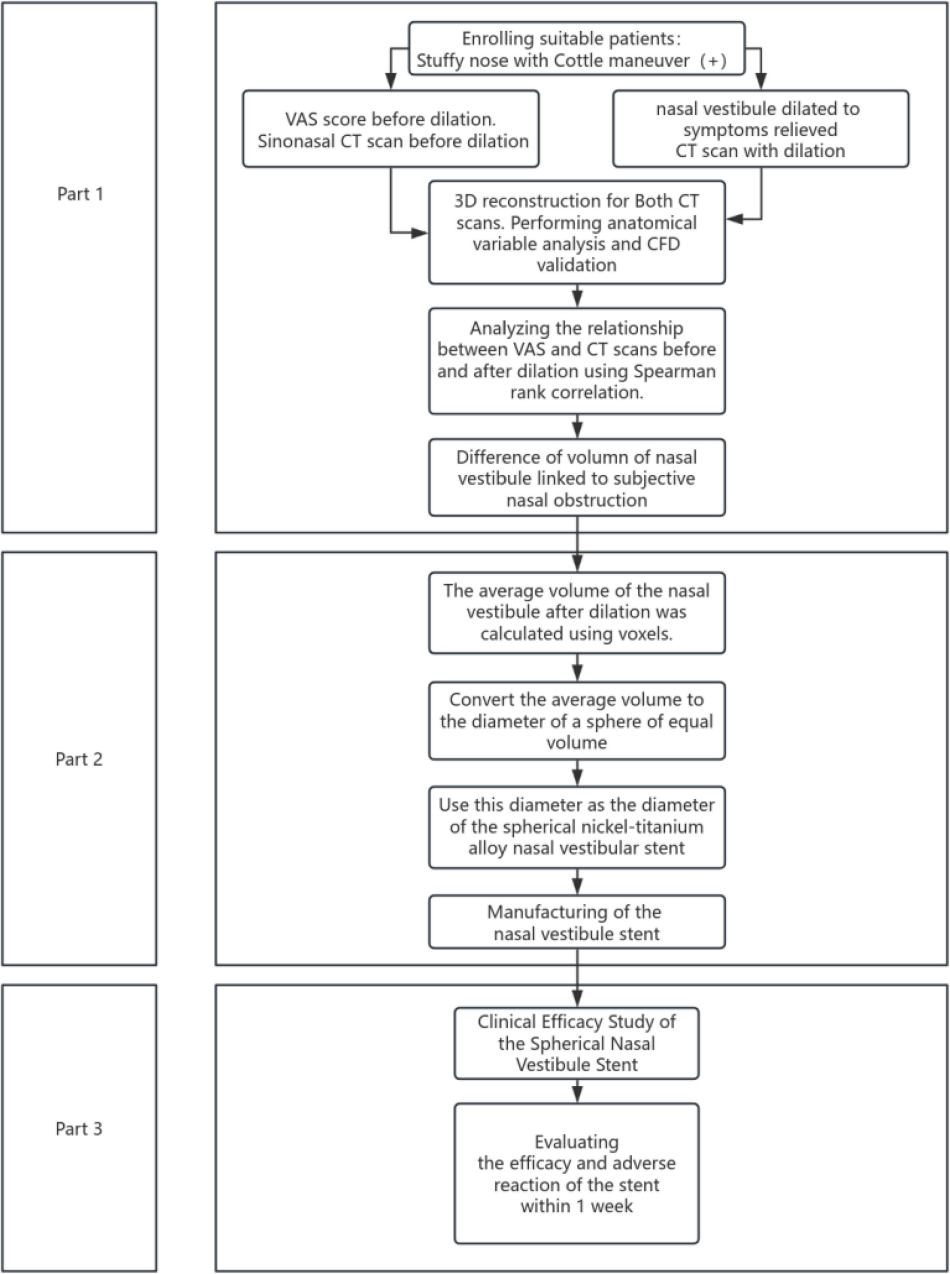
Study flowchart.

This part used a single-arm design. Participants were selected from Part 1 on a voluntary basis. Prior to stent placement, patients completed the NOSE questionnaire, acoustic rhinometry, and rhinomanometry. Acoustic rhinometry and rhinomanometry were repeated immediately following stent placement to assess changes in nasal patency and airflow dynamics. Patients wore the stent for at least 8 h daily. Follow-up NOSE assessments were conducted on days 1, 3, and 7. Outcomes included subjective symptoms (NOSE score) and objective measures (rhinomanometry, acoustic rhinometry). The nasal-related adverse reaction questionnaire, non-nasal-related adverse reaction questionnaire, and Western Nasal Dilation Tolerance Scale were employed to assess the safety and tolerability of the nasal stent. Flowchart of this study was shown in Figure 2.

### Statistical analysis

To evaluate the relationship between nasal vestibular volume changes and symptoms, Spearman correlation analysis was performed between volume change parameters and VAS scores. Paired t-tests compared CFD-derived hydrodynamic parameters before and after dilation; *p* < 0.05 was considered significant. All analyses used SPSS 27.0, with *α* = 0.05.

For stent efficacy, sample size was calculated using Simon’s two-stage design. Paired t-tests analyzed NOSE scores, nasal resistance, and volume. Cohen’s d was computed for effect size. Adverse event rates were reported as percentages. All tests used SPSS 27.0; p < 0.05 indicated significance.

### Results

#### Part 1: 3D reconstruction results before and after nasal vestibular dilation

**Figure 3:**
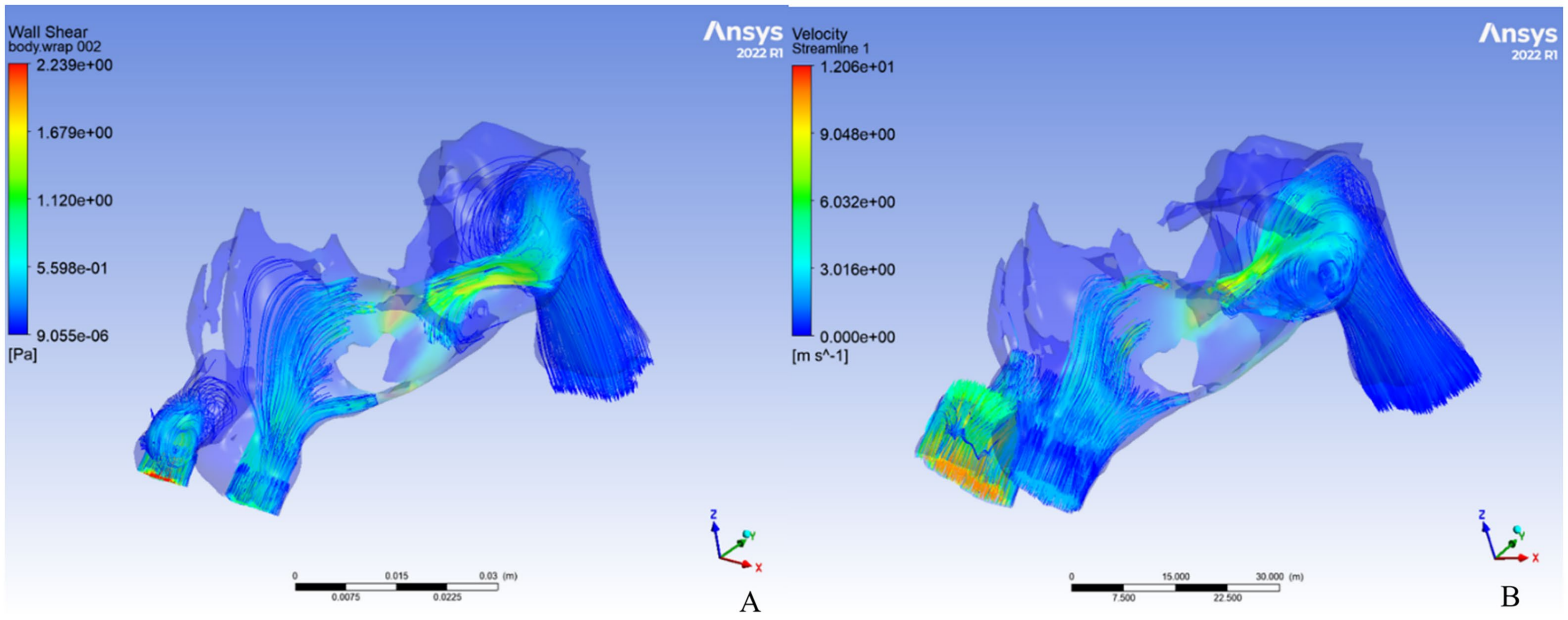
CFD analysis of a three-dimensional reconstruction model and streamline illustrations: **(A)** Before anterior rhinoscopy dilation; **(B)** After anterior rhinoscopy dilation.

A total of 99 patients (70 males, 29 females; mean age 36.8 ± 11.0 years) were enrolled. 3D models were generated from sinonasal CT scans, and nasal vestibular volume parameters were calculated. Spearman correlation analysis was performed between VAS scores for nasal obstruction and volume change parameters; results are in Table 1. Hydrodynamic parameters, including average flow velocity, pressure difference, and wall shear stress, were computed to assess changes before and after dilation; results are in Figure 3 and Table 2.

**Table 1:** Volumes and related parameters of nasal vestibule.

Index	Average (𝑥±s)	R^2^
Volume of left side nasal vestibule before dilation (mm^3^)	1200.44 ± 324.65	
Volume of left side nasal vestibule after dilation (mm^3^)	1952.06 ± 474.53	
Volume of right side nasal vestibule before dilation (mm^3^)	1222.48 ± 326.25	
Volume of right side nasal vestibule after dilation (mm^3^)	1955.21 ± 435.60	
Difference of volume of left side nasal vestibule before and after dilation (mm^3^)	774.59 ± 357.05	0.374
Rate of volumetric change of left side nasal vestibule (%)	71.78 ± 39.49	0.405
Difference of volume of left side nasal vestibule before and after dilation (mm^3^)	746.60 ± 333.13	0.462
Rate of volumetric change of right side nasal vestibule (%)	68.92 ± 39.48	0.555
Left–right difference in nasal vestibule volume before dilation (mm3)	162.94 ± 128.97	0.041
Volumetric rate of left–right difference in nasal vestibule before dilation (%)	6.64 ± 4.83	0.095

**Table 2:** Paired *t* test of CFD outcomes before and after stent placement.

Index	Before dilation	After dilation	*P*
Average nasal airflow velocity(m/s)	1.47 ± 0.63	1.50 ± 0.63	0.231
Average nasal air pressure(Pa)	1.11 ± 1.40	1.07 ± 1.40	0.170
Average nasal wall shear stress(Pa)	0.17 ± 0.44	0.05 ± 0.08	0.014*

*p < 0.05.

CFD results derived from the 3D models revealed no significant differences in average velocity or pressure (*p* > 0.05). However, the average wall shear stress decreased significantly after dilation (t = 2.537, *p* = 0.014).

#### Part 2: Clinical efficacy of the spherical nasal vestibular stent

In our single-arm clinical trial, the sample size was calculated using Simon’s two-stage design (one-sided *α* = 0.05, power = 80%), with planned enrollment of 28–42 patients. Ultimately, a total of 31 patients from Part 1 participated in this study on a voluntary basis. The study is registered at Clinicaltrials.gov (NCT05243147).

##### Subjective symptom evaluation

**Figure 4:**
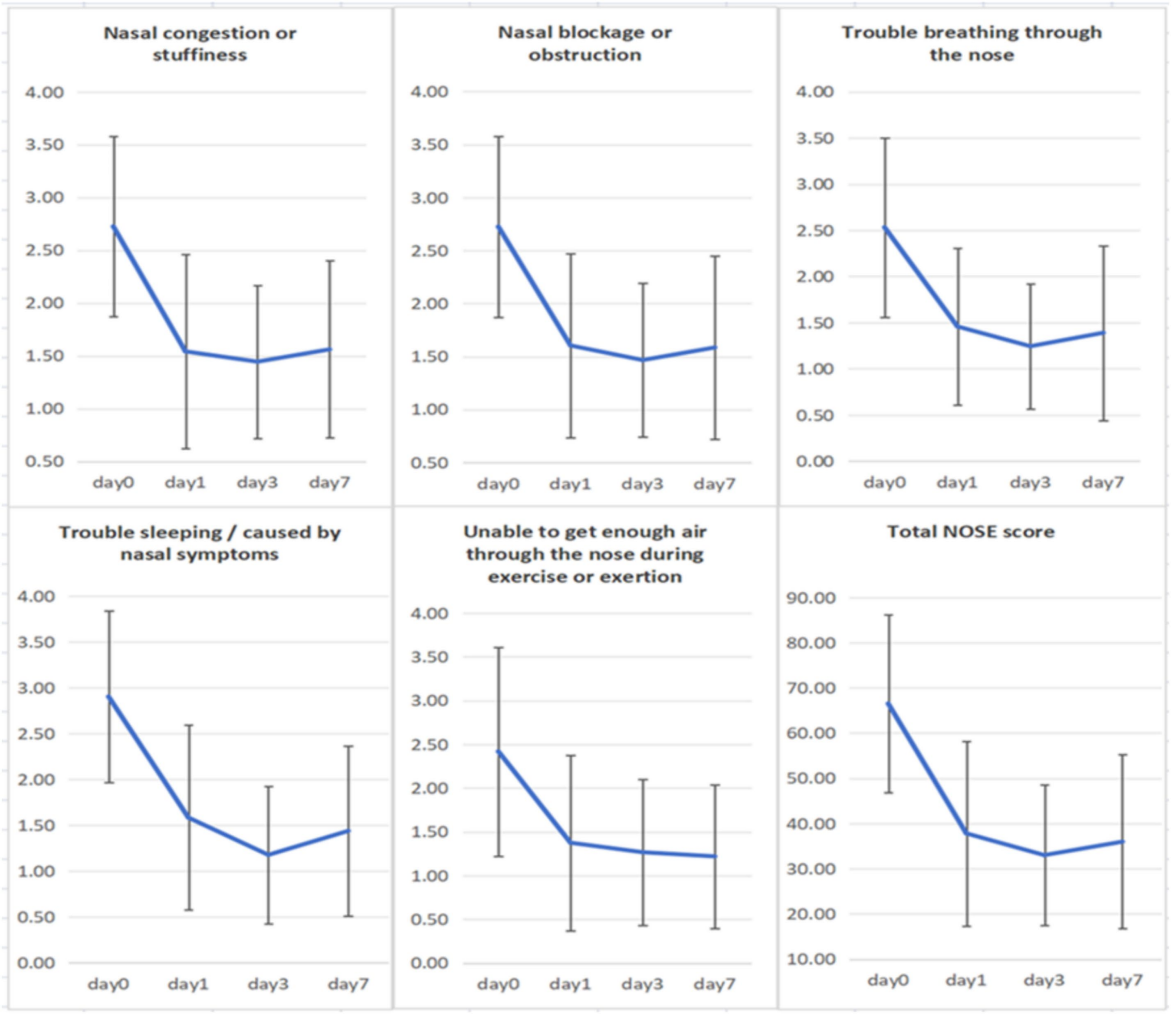
NOSE score (Day 0 refers to pre-stent placement; days 1, 3, and 7 refer to the 1st, 3rd, and 7th day post-stent placement).

The NOSE scale was used to assess stent efficacy in improving nasal ventilation. Scores from 31 subjects before and after stent use were compared using a paired t-test; effect size was calculated. Results are shown in Figure 4 and Table 3.

**Table 3:** Paired *t* test and effect size of NOSE score before and after stent placement.

Index	Day0 vs. Day1^#^		Day0 vs. Day3^#^		Day0 vs. Day7^#^	
	*P*	Cohen’s d value	*P*	Cohen’s d value	*P*	Cohen’s d value
Nasal congestion or stuffiness	<0.01	0.88	<0.01	1.003	<0.01	0.852
Nasal blockage or obstruction	<0.01	0.86	<0.01	1.056	<0.01	0.815
Trouble breathing through the nose	<0.01	0.742	<0.01	0.914	<0.01	0.669
Trouble sleeping / caused by nasal symptoms	<0.01	1.016	<0.01	1.34	<0.01	1.101
Unable to get enough air through the nose during exercise or exertion	<0.01	0.664	<0.01	0.734	<0.01	0.724
Total NOSE score	<0.01	1.015	<0.01	1.212	<0.01	1.037

^#^Day 0 refers to pre-stent placement; Days 1, 3, 7 refer to the 1st, 3rd, and 7th day post-stent placement.

The mean baseline NOSE score was 58.55 ± 20.62. Following one day of stent use, the score decreased to 33.68 ± 19.50, further declined to 30.00 ± 16.58 by day three, and remained stable at 30.00 ± 14.39 by day seven. Effect size analysis using Cohen’s d revealed a mean reduction of 26.15 (d = 1.015) on day one, 30.11 (d = 1.212) on day three, and 25.61 (d = 1.037) on day seven.

##### Objective indicator evaluation

Rhinomanometry and acoustic rhinometry were performed before and after stent placement. Results are presented in Table 4.

**Table 4:** Results of nasal resistance and acoustic rhinometry changes before and after stent placement.

Index	Before sent placement	After sent placement	*p*
Left side inhaling nasal resistance	0.36 ± 0.04	0.24 ± 0.02	<0.001**
Left side exhaling nasal resistance	0.24 ± 0.04	0.23 ± 0.02	<0.001**
Right side inhaling nasal resistance	0.40 ± 0.34	0.28 ± 0.16	0.005**
Right side exhaling nasal resistance	0.29 ± 0.14	0.25 ± 0.07	0.031*
Total volume of left nasal cavity	6.69 ± 1.08	7.85 ± 0.92	<0.001**
Left nasal valve area	0.77 ± 0.24	1.11 ± 0.35	<0.001**
Total volume of right nasal cavity	5.88 ± 1.27	7.31 ± 1.90	<0.001**
Right nasal valve area	0.68 ± 0.11	1.01 ± 0.12	<0.001**

*p < 0.05; **p < 0.01.

##### Adverse event evaluation

**Figure 5:**
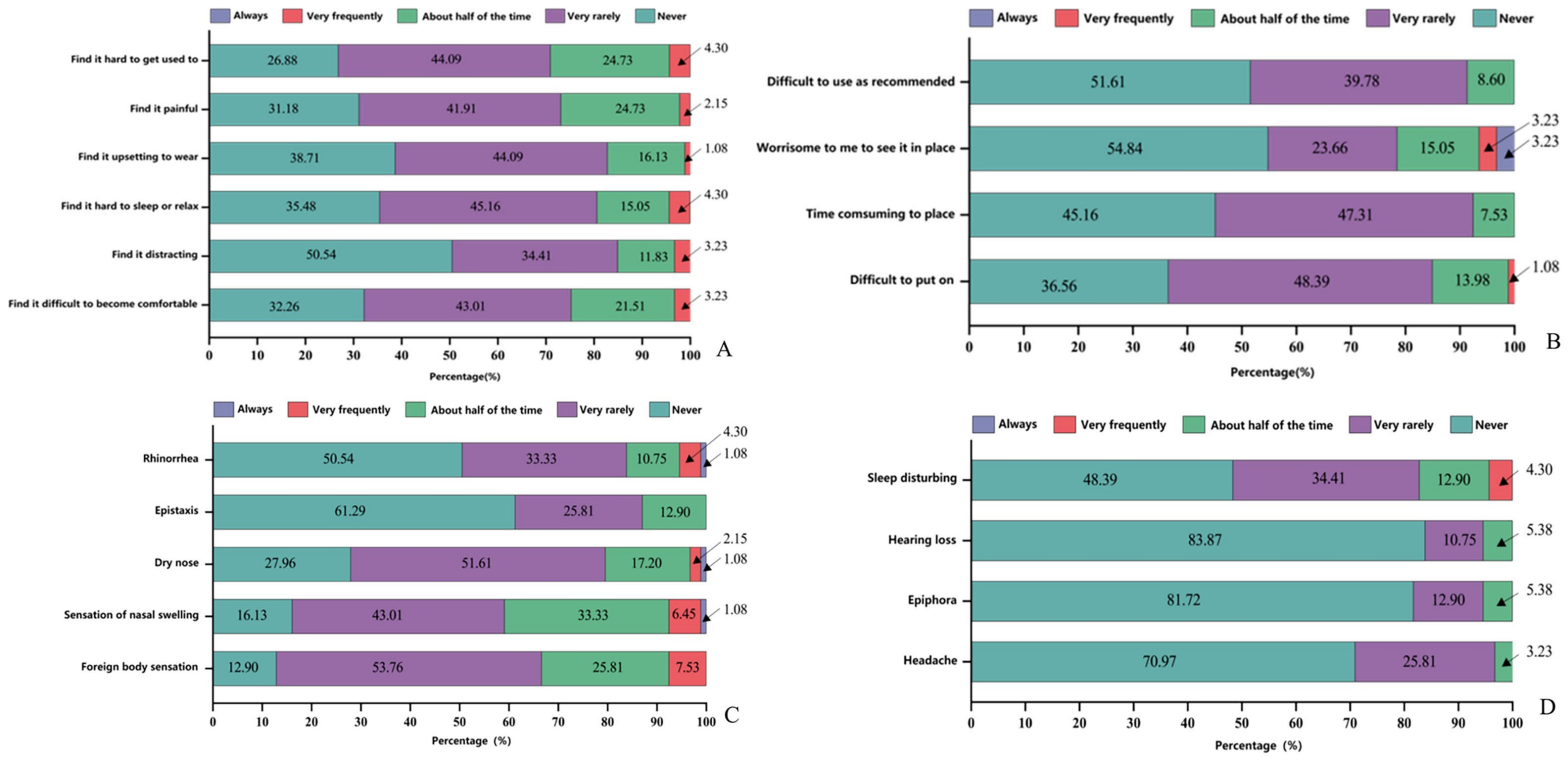
Adverse event incidence. **(A,B)** Stent-wearing experience (assessed by the Western Nasal Dilation Tolerance Scale). **(C)** Nasal-related adverse reactions. **(D)** Non-nasal-related adverse reactions.

Patients completed an adverse event questionnaire at 1, 3, and 7 days after stent use. Incidence is shown in Figure 5.

### Discussion

This study aimed to design a universal nasal vestibular stent based on anatomical changes. To establish the rationale, we analyzed the correlation between nasal vestibular volume changes and obstruction symptoms in patients with a positive anterior rhinoscopy test. Using 3D reconstruction from sinonasal CT scans, we measured nasal vestibular volumes. These results indicate that patients with smaller baseline volumes and greater dilation experience more severe symptoms. Thus, the stent should be designed to increase nasal vestibular volume by enhancing local spatial expansion. We also assessed bilateral symmetry effects. No significant correlation was found between symptom scores and absolute volume difference or asymmetry index, suggesting asymmetry is not a primary factor. Therefore, for unilateral stenosis, stent placement may be needed only on the affected side without compromising contralateral airflow.

CFD serves as an objective indicator of nasal ventilation function (14, 15). Unlike our previous study focusing only on the nasal vestibule (16), this analysis included the entire nasal cavity and both sides simultaneously. Average wall shear stress, decreased significantly after dilation, dropping by 0.012 Pa, more than half of pre-dilation levels. Wall shear stress is the tangential frictional force at the wall surface caused by fluid viscosity and near-wall velocity gradient, it is proportional to both viscosity and velocity gradient. This parameter is critical in engineering and biomedical applications and reflects airway resistance in nasal CFD (17–19). The marked reduction in wall shear stress indicates improved nasal hydrodynamics and ventilation. Thus, designing a nasal vestibular stent based on volume parameters to relieve obstruction symptoms is feasible.

Currently, clinical nasal vestibular stents fall into two categories: external and internal dilators. Internal types include intranasal stents, nasal clips, and septal stimulators (8, 9, 20). External dilators are limited by visible strips and adhesive-related skin inflammation. Internal dilators are typically made of silicone (9, 12) or stainless steel (21). Silicone may lose elasticity over time, compromising long-term dilation. Metal devices can cause discomfort, poor biocompatibility, and mucosal irritation due to direct contact with nasal tissues. In contrast, this study adopts a novel approach focused on increasing overall nasal vestibular volume. By optimizing material and design, we developed a spherical nitinol stent. Nitinol provides excellent biocompatibility and shape memory. The spherical structure better matches the nasal vestibule’s anatomy, enhancing airflow through volume expansion while minimizing mucosal irritation.

**Figure 6:**
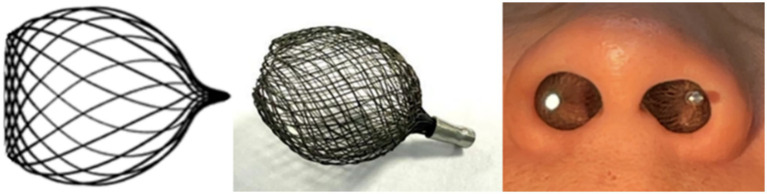
Nasal vestibular stent and its placement within patient’s nasal vestibule.

Our prior research (16) shows that spherical dilation offers optimal mechanical support in the nasal vestibule and the stents were shown in Figure 6. The nasal vestibule is narrower at the anterior naris and nasal valve but wider centrally, making a spherical design well-suited to its natural shape. A sphere’s continuous curved surface distributes external forces evenly, as the normal at any point directs toward the center, and fluid pressure on a sphere is uniform. Based on post-dilation volume measurements, we designed a 15.5 mm spherical nasal stent. To balance comfort and ventilation, the stent has an open posterior end, positioning it close to, but not touching the nasal valve mucosa. While it does not directly enlarge the internal nasal valve cross-section, it indirectly improves airflow by supporting lateral alar tissue, effectively increasing both valve area and overall vestibular volume. We selected nitinol for its biocompatibility, corrosion resistance, shape memory, and super elasticity, properties well-established in clinical use (22). Our group previously used woven nitinol stents post-septal surgery, observing strong support, reduced discomfort, and no complications (11). Unlike existing bilateral nasal strips, stents, or clips, which may cause discomfort on the unaffected side in unilateral cases, our stent is worn unilaterally without impairing contralateral airflow.

Subjective symptom improvement was evaluated using the NOSE score. The NOSE score demonstrated a significant reduction from baseline (before stent use) to post-wearing, and remained stable without increase as the duration of wear extended, indicating rapid and substantial relief from nasal obstruction within the first day, with sustained efficacy over time. Effect size analysis using Cohen’s d also confirmed large effects. These results demonstrate that the spherical nasal vestibular stent effectively alleviates nasal obstruction. For objective assessment, rhinomanometry and acoustic rhinometry were used. Following stent placement, nasal resistance during both inhalation and exhalation significantly decreased in both nostrils (*p* < 0.01), while overall nasal volume and the nasal valve area showed significant increases (*p* < 0.001). These findings indicate a substantial improvement in nasal ventilation function.

The safety profile and user experience associated with the nasal vestibule stent were also rigorously evaluated. Safety was assessed in terms of both nasal and non-nasal adverse reactions, while subjective tolerance and wearing experience were evaluated using the Western Nasal Dilation Tolerance Scale (21). The Western Nasal Dilation Tolerance Scale is a validated assessment instrument specifically developed for the evaluation of nasal stents in research and clinical practice, designed to capture patient-reported feedback on device use. It primarily assesses comfort during wear and the presence of adverse events, encompassing dimensions such as user experience, ease of use, potential discomfort, and associated complications. Nasal adverse reactions were mostly mild to moderate: nasal dryness occurred in 72.04% of reports (51.61% mild, 20.43% moderate or higher), foreign body sensation or swelling in 70–80%, and minor bleeding in 12.90%, with no active bleeding. Only one case reported severe rhinorrhea and swelling, which resolved after temporary discontinuation. Non-nasal systemic symptoms were rare: 51.61% reported mild to moderate sleep interference, and headache and tearing were infrequent (70.97–83.87% reported no effect), with no serious systemic events. Regarding wear tolerance, 73.18% rarely or never experienced pain, though 26.88% reported discomfort during rest or sleep, and 21.51% expressed concerns about appearance. Most users (84.95–92.47%) found the stent easy and quick to insert, indicating good usability. Overall, the stent showed favorable safety and tolerability, with primarily mild, reversible local reactions. These findings support design improvements, such as surface hydration, and better patient education to enhance adaptation.

This study still has certain limitations in terms of experimental design and aesthetic craftsmanship. From the perspective of experimental design, future research should include more subjects of different races and genders to reduce potential racial and gender biases. At the same time, the sample size should be expanded, the follow-up period should be prolonged, and a randomized controlled trial design should be adopted to directly compare with existing mainstream therapies, thereby providing higher-level evidence-based medical evidence for the clinical application of spherical nasal vestibular stents. In terms of aesthetics and craftsmanship, subsequent work can focus on optimizing the weaving process to improve the appearance design of the stent, promoting the development of personalized customization solutions, and further enhancing wearing comfort and user experience.

### Conclusion

The spherical nasal vestibular stent, designed based on nasal vestibular volume parameters, can increase the overall nasal volume and nasal valve area in patients with nasal obstruction, significantly alleviating nasal obstruction symptoms in patients with a positive anterior rhinoscopy test. It demonstrates good safety and can serve as an effective non-surgical treatment option for clinical use.

## Data Availability

The original contributions presented in the study are included in the article/supplementary material, further inquiries can be directed to the corresponding authors.
